# Optimizing in-hospital mortality predictive models in ACS patients: QTc prolongation and machine learning approaches

**DOI:** 10.1186/s43044-025-00639-x

**Published:** 2025-04-19

**Authors:** Hamrish Kumar Rajakumar

**Affiliations:** Government Medical College, Omandurar Government Estate, Chennai, 600 002 Tamilnadu India

To the Editor,

I read with great interest the article by El Amrawy et al. regarding the use of QTc interval prolongation and machine learning (ML) to predict in-hospital mortality among acute coronary syndrome (ACS) patients [1]. This study highlights the potential of ML in enhancing clinical decision making. The early prediction model (EPM) using accessible clinical and ECG data is practical and cost-effective. However, several points warrant further discussion to refine the study’s applicability and potential clinical impact.

Although the authors addressed data imbalance by resampling and class balancing techniques, these methods may fall short of fully capturing the characteristics of the minority class (nonsurvivors). Exploring synthetic minority oversampling technique (SMOTE) or AdaBoost could add depth to the analysis [2, 3]. Furthermore, training other ML models, such as neural networks (NN), extreme gradient boosting (XGBoost), light gradient boosting machine (LGBM), gradient boosting machine (GBM), and support vector machines (SVMs), could provide additional comparative analysis and refine model selection for optimal clinical application.

Interpretability remains a significant hurdle in deploying ML models in clinical practice. Techniques such as SHAP (Shapley Additive exPlanations) or LIME (Local Interpretable Model-Agnostic Explanations) could make the models more acceptable to clinicians by clarifying how variables affect predictions [4, 5]. A crucial aspect overlooked in this study is the lack of clarity regarding the use of bootstrapping and hyperparameter tuning, which can improve model generalizability.

The use of a QTc interval cutoff of 450 ms on the basis of Bazett’s formula raises another issue. This formula is known to perform poorly at heart rate extremes [6]. Employing alternative correction methods such as Fridericia or Hodges might have improved the accuracy of risk stratification [7]. Additionally, considering sex-specific QTc thresholds could have refined the predictive capability further.

The approach to handling missing data also warrants scrutiny. Relying on simple mean and mode imputation may oversimplify the dataset and risk introducing bias. Advanced techniques such as multiple imputation by chained equations (MICE) could have improved the strength of the models by preserving the underlying data distribution, and the k-nearest neighbors method could further improve data completeness [8]. Another limitation lies in the study’s focus on in-hospital mortality. Expanding the model to predict long-term outcomes such as recurrent myocardial infarction, heart failure, or overall mortality could make it more relevant for comprehensive patient management.

This study incorporates GRACE and TIMI scores, but a more in-depth analysis of how these traditional risk scores interact with ML-based predictions and assessing whether a combined integrated approach (ML with conventional risk scores) offers superior predictive value could provide further clinical insights and improve risk stratification strategies. While the study highlights QTc prolongation as an independent risk predictor, incorporating additional ECG parameters such as T-wave morphology or heart rate variability might offer a more detailed understanding of myocardial ischemia and its prognostic implications. These complementary features could strengthen risk stratification when combined with QTc.

In conclusion, the work of El Amrawy et al. is a valuable step toward integrating ML into clinical practice for ACS management. Addressing these areas of improvement could further strengthen the study’s relevance and maximize its potential impact.

## Data Availability

No datasets were generated or analyzed during the current study.

## Abbreviations

QTc: Corrected QT interval
ML: Machine Learning
ACS: Acute coronary syndrome
EPM: Early prediction model
ECG: Electrocardiogram
SMOTE: Synthetic minority oversampling technique
AdaBoost: Adaptive boosting
NN: Neural networks
XGBoost: Extreme gradient boosting
LGBM: Light gradient boosting machine
GBM: Gradient boosting machine
SVMs: Support vector machines
SHAP: Shapley Additive exPlanations
LIME: Local Interpretable Model-Agnostic Explanations
MICE: Multiple imputation by chained equations
GRACE: Global registry of acute coronary events
TIMI: Thrombolysis in myocardial infarction
